# Comprehensive genome-wide analysis of wheat xylanase inhibitor protein (XIP) genes: unveiling their role in Fusarium head blight resistance and plant immune mechanisms

**DOI:** 10.1186/s12870-024-05176-4

**Published:** 2024-05-27

**Authors:** Juan Lin, Shuang Ruan, Qi Guo, Yonglin Zhang, Mengyuan Fang, Tiantian Li, Gan Luo, Zhuangbo Tian, Yi Zhang, Erwin Tandayu, Can Chen, Jie Lu, Chuanxi Ma, Hongqi Si

**Affiliations:** 1 College of Agronomy, Anhui Agricultural University, Hefei, 230036 China; 2 Key Laboratory of Wheat Biology and Genetic Improvement on Southern Yellow and Huai River Valley, Ministry of Agriculture and Rural Affairs, Hefei, 230036 China; 3 Faculty of Science and Engineering, Southern Cross University, Lismore, NSW 2480 Australia

**Keywords:** Plant pathogen resistance, Gene expression analysis, Fungal disease management, Wheat genomics, Plant hormone signaling, Crop breeding strategies

## Abstract

In this comprehensive genome-wide study, we identified and classified 83 Xylanase Inhibitor Protein (XIP) genes in wheat, grouped into five distinct categories, to enhance understanding of wheat's resistance to Fusarium head blight (FHB), a significant fungal threat to global wheat production. Our analysis reveals the unique distribution of XIP genes across wheat chromosomes, particularly at terminal regions, suggesting their role in the evolutionary expansion of the gene family. Several XIP genes lack signal peptides, indicating potential alternative secretion pathways that could be pivotal in plant defense against FHB. The study also uncovers the sequence homology between XIPs and chitinases, hinting at a functional diversification within the XIP gene family. Additionally, the research explores the association of XIP genes with plant immune mechanisms, particularly their linkage with plant hormone signaling pathways like abscisic acid and jasmonic acid. *XIP-7A3*, in particular, demonstrates a significant increase in expression upon FHB infection, highlighting its potential as a key candidate gene for enhancing wheat's resistance to this disease. This research not only enriches our understanding of the XIP gene family in wheat but also provides a foundation for future investigations into their role in developing FHB-resistant wheat cultivars. The findings offer significant implications for wheat genomics and breeding, contributing to the development of more resilient crops against fungal diseases.

**Supplementary Information:**

The online version contains supplementary material available at 10.1186/s12870-024-05176-4.

## Background

Fusarium head blight (FHB) disease in wheat, caused by the fungus *Fusarium graminearum* (*F. graminearum*), leads to premature blighting and ultimately results in shrunken kernels, contributing to significant yield losses of 10-70% [1, 2]. The grain also becomes unsafe for human consumption due to accumulated mycotoxins produced during infection. Currently, the most cost-effective and eco-friendly strategy to mitigate FHB outbreaks involves cultivating cultivars that exhibit stable and long-lasting resistance to FHB [3]. However, breeding for durable FHB-resistant wheat varieties is challenging owing to the complex and polygenic nature of the FHB-resistant trait. Traditional breeding methods often capture only a subset of the necessary resistance genes, resulting in cultivars with incomplete resistance potential and, consequently, only moderate resistance [4, 5]. The publication of whole wheat genome sequence has opened up many avenues for detailed and targeted research into the genes associated with FHB resistance [6, 7]. With this genomic resource, advanced genetic techniques can be conducted to expand our knowledge of the trait and aid in the breeding of FHB-resistant varieties.

Xylan, a type of polysaccharide, is a major component and is primarily found in plant cell wall [8]. In the wheat cultivar Sumai3, the downregulation of genes involved in the xylan catabolic process is noted to contribute to its resistance against pathogens [9]. During an invasion by pathogenic fungi such as *F. graminearum*, these fungi secrete xylanases [10], which break down xylans, compromising the wheat cell wall integrity and facilitating further fungal invasion and spread [11, 12]. Xylanase inhibitors (XIs) in wheat serve the critical function of neutralizing and inhibiting these exogenous xylanases [13]. In wheat, three distinct types of XIs have been identified based on their structural characteristics, namely thaumatin-like xylanase inhibitor (TLXI) type inhibitors [14], *Triticum aestivum* xylanase inhibitor (TAXI) [15] and xylanase inhibiting protein (XIP) [16]. Each type of XI possesses unique domains that aid in their identification. TLXI shares significant homology with thaumatin-like proteins complicating the precise identification of TLXI family members [13, 17]. For TAXI classification, both the xylanase inhibitor N-terminal and the Xylanase inhibitor C-terminal are used [18], whereas for XIP classification the glycoside hydrolase family 18 (GH18) is used. However, to date, a comprehensive identification of wheat XIP gene members remains lacking.

Several features suggest that XIs play a pivotal role in host defense mechanisms. Notably, they exhibit both sequence and structural homology with various pathogenesis-related proteins, and their expression is typically upregulated under salicylic acid, jasmonic acid (JA), methyl jasmonate treatment and stress conditions such as wounding and FHB infection [13, 18]. For instance, specific members of the TAXI gene family, including *TaXI-IIA*, *TaXI-III* and *TaXI-IV* were significantly induced by FHB infection in wheat spikes [18]. Transgenic durum wheat plants overexpressing *TAXI-III* demonstrated a reduction in disease symptoms caused by FHB [19]. A synergistic resistance effect was observed when TAXI-III and PvPGIP2 -a polygalacturonase-inhibiting protein from *Phaseolus vulgaris*, were combined in durum wheat, leading to increased disease resistance against FHB compared to lines containing either only TAXI-III or PvPGIP2 [20]. Similarly, rice XIs are also induced by pathogen infection. Overexpression of *Os-XIP* or *RIXI* in rice resulted in enhanced disease resistance to fungi *Pyricularia oryzae* [21–23]. Additionally, the xylanase inhibitor-like protein from sorghum inhibited the mycelial growth of *Fusarium oxysporum*, demonstrating its antifungal activity [24]. The insights underscore the potential of XIs in fungal disease resistance and should be harnessed through genomic and breeding strategies. Previous research thoroughly reported the expression pattern of wheat *TAXIs*. In the promoter region of the wheat TAXI gene, a recent study identified several cis-acting elements linked to biotic stresses (FHB, powdery mildew, and stripe rust infestation), abiotic stresses (drought, high and low temperatures), and phytohormones (methyl jasmonate, salicylic acid, abscisic acid (ABA), gibberellin, auxin, and ethylene) [18]. However, recent information on the expression patterns of wheat XIP genes is limited, and the response of wheat XIP genes to FHB infection remains somewhat ambiguous.

In our research, a comprehensive search of the wheat genome database (IWGSC RefSeq v1.1) led to the identification of 83 XIP genes, including the four known isoforms encoded by genes such as *XIP-I*, *XIP-III* and *XIP-R* (*XIP-R1* and *XIP-R2*) [16, 25, 26]. Utilizing in silico analyses of publicly available RNA-seq data from various stages of FHB infection, we focused on a subset of XIP genes that showed potential association with FHB response. This approach allowed us to shortlist 20 candidate genes exhibiting upregulation post-FHB infection, suggesting their involvement in wheat's defense mechanisms against the disease. This research serves as a foundational step for future investigations into wheat's defense mechanisms against FHB. To validate the biological significance of these findings, targeted analyses were conducted emphasizing the potential of specific XIP genes, such as *XIP-4D1* (also known as *XIP-I*) and notably *XIP-7A3*, which emerged as promising candidates due to their substantial upregulation in response to FHB infection, particularly in resistant cultivars. Our study combines publicly available data analyses with targeted validation of XIP genes associated with FHB infection, laying the groundwork for future functional genomic studies and the potential exploitation of these findings in breeding programs aimed at enhancing FHB resistance through precise gene targeting.

## Methods

### Identification of XIP gene family members in wheat

To systematically identify the XIP gene family members in the wheat genome, the complete genome DNA sequence, CDS, Pep and annotation files for wheat were sourced from the Ensemble Plants database (https://plants.ensembl.org/index.html). The associated Pfam ID for the XIP gene was retrieved from the Uniprot database (https://www.uniprot.org/) followed by the acquisition of the XIP Hidden Markov model (HMM) from the Pfam website [27] (http://pfam.xfam.org/). Using the TBtools software version 2.012 [28], the “Simple HMM Search” was employed, and the resulting output was saved. XIP sequences from wheat and other species were retrieved from the National Center for Biotechnology Information (NCBI) (https://www.ncbi.nlm.nih.gov/Structure/cdd/wrpsb.cgi). These sequences were then compared against the whole wheat genome protein sequence by BLASTp. Sequences identified through both HMM search and BLASTp were combined, ensuring the removal of duplicates. For the identification of structural domains, both the Pfam website and the Batch CD-search mode on the NCBI were used. Genes lacking or with incomplete structural domains were excluded, ensuring the final list comprised high-confidence wheat XIP gene family members.

### Protein physicochemical properties, subcellular localization and signal peptide prediction

To determine the molecular weight, isoelectric point, and instability coefficient of the XIP gene family members, the ExPAsy online tool [29] (https://web.expasy.org/protparam/) was utilized. Subcellular localization was inferred using the Plant-mPLoc database on the Cell-PLoc 2.0 online platform (http://www.csbio.sjtu.edu.cn/bioinf/Cell-PLoc-2/) [30]. Signal peptide prediction was performed via the SignalP website (https://services.healthtech.dtu.dk/service.php?SignalP-5.0), and results were obtained in batch format.

### Phylogenetic analysis and classification

Using the XIP-type protein sequences from wheat, durum wheat, and rice sourced from the NCBI database, an initial alignment was performed using ClustalW comparison with default parameters. Evolutionary trees were constructed using Molecular Evolutionary Genetics Analysis Version 11 (MEGA11) v.11.0.11 software [31]. Neighbor-joining method was employed for tree construction, utilizing p-distance, bootstrap method with 1000 duplicates, partial deletion, and site coverage cutoff of 50%. The resulting phylogenetic tree was visualized using the Interactive Tree Of Life online tool (https://itol.embl.de/) with tree topology, subsequently used for classification.

### Analysis of gene structure, protein motifs, collinearity and chromosome localization

Gene structures were derived from wheat annotation files. Identification of conserved protein motifs within the XIP gene family was conducted using the MEME online tool [32] (http://meme-suite.org/) targeting 20 motifs with default parameters. Each predicted motif has been annotated through a search of the Pfam database. The resulting motifs and gene structures were visualized using the “Gene Structure View (Advanced)” function of TBtools. Collinearity of wheat genes was carried out depicted in an advanced circos plot generated using TBtools. The physical location of the predicted wheat XIP genes on wheat chromosomes was mapped using the “Gene Location Visualize from GTF/GF” function in TBtools.

### *Cis*-acting element analysis and visualization

The 2000 base pairs (bp) genomic sequence upstream of the predicted XIP gene coding start sequence was extracted from the Wheat Chinese Spring Reference Genome using TBtools, designating this sequence as the gene’s promoter region. Cis-acting elements within this promoter region were identified via the PlantCARE online tool (http://bioinformatics.psb.ugent.be/webtools/plantcare/html/). The resulting data was compiled and visualized as cis-acting elements using TBtools.

### RNA-Seq data analysis

To investigate the expression patterns of wheat XIP genes under FHB infection, wheat RNA-seq data [33] for XIP genes were downloaded from the wheat-expression database (http://www.wheat-expression.com/). Expression profiles from the *Triticum aestivum* RNA-seq Database (http://ipf.sustech.edu.cn/pub/wheatrna/) were visualized using TBtools.

### Multiple sequence comparison and homology analysis

Sequence alignment was performed using DNAMAN (V6.0, Lynnon Biosoft, USA). Amino acids with similarity were colored, based on their homology levels: ≥33% in yellow, ≥50% in blue, ≥75% in pink, 100% in black, and less than 33% in white. The sequence identity of XIPs, both at the nucleotide and amino acid levels, was determined using the ClustalW method in the MegAlign program of Lasergene V7.1.0 software. The resulting data was structured, with color gradients applied to signify varying values.

### GO and KEGG analysis

Functional annotations of the genes were performed using the GO: Gene Ontology Resource (http://geneontology.org/) and KEGG: Kyoto Encyclopedia of Genes and Genomes (https://www.genome.jp/kegg/). Visualization of the results was carried out using the plotting tool available at a bioinformatics online tool (https://www.bioinformatics.com.cn/plot_basic_go_pathway_circlize_plot_140).

### Functional and phylogenetic analysis of homologous genes

Homologous genes from rice and Arabidopsis were identified using the wheatomic platform (http://202.194.139.32/homologtools/index.html). Evolutionary trees for these homologous genes from wheat, rice, and *Arabidopsis thaliana* were constructed using MEGA11 v11.0.11 software [31].

### Plant material and qRT-PCR analysis

Three distinct wheat cultivars, namely Wangshuibai (WSB), Annong1589 (AN1589) and Annong8455 (AN8455), exhibiting varying FHB resistance, were selected and planted at the Hefei High-tech Agricultural Park in Anhui Province. At the heading stage of wheat, a 10 μl spore suspension of *F. graminearum* was administered into each floret of the central spikelet using single flower inoculation. Post-infection, samples were collected at intervals of 12 h, 24 h, 48 h, 72 h, and 96 h after infection, with uninfected spikelets serving as controls. These samples were immediately frozen in liquid nitrogen and stored at -80℃. This process was replicated three times for biological accuracy. To investigate the disease progression in each cultivar post-FHB infection, 10 spikes from each cultivar were randomly chosen, and the average severity was evaluated after 21 days (Table S7) using the formula: Average severity = ∑ (Number of diseased spikes at all levels × Representative value at all levels) / Total number of investigated spikes. Specific grading and calculation were followed according to Zhang et al. [34].

The qRT-PCR experimental method used in this study was published by Hu et al. [35]. Using Premier Primer v5.0 software, we designed specific primers at positions where the sequences of coding regions differed (Table S1). We used the *Actin* gene of wheat as the internal reference gene and all primers were synthesized by Shanghai Biotech Biological Co. Three replicate experiments were designed for each reaction. The relative expression levels were analyzed using the 2^-ΔΔCT^ method [ΔΔCT = (CTtarget/F.g-CTactin/F.g) - (CTtarget/control-CTactin/control)]. ' F.g ' indicates 12 h, 24 h, 48 h, 72 h and 96 h samples infected with *F. graminearum*, while ' control ' refers to 0 h samples without FHB infection.

## Results

### Genome-wide identification and phylogenetic analysis of XIP family members

In our genome-wide exploration, we identified a total of 83 XIP genes in wheat. These proteins were systematically named from XIP-1A1 to XIP-Un2 (‘Un’ represents XIP genes whose chromosomal locations in wheat are unknown), based on their chromosomal position and sequence (Table S2). The characteristics of these proteins varied, with sequence lengths ranging from 191-739 amino acids (aa), gene lengths between 576-2220 bp, and molecular weights from 20.270-79.77466 kD. Their instability coefficients spanned 15.11-57.27, and the isoelectric points were between 4.1-9.87 (Table S2). Notably, 50 XIP exhibited acidic theoretical isoelectric points, while the remaining 33 were basic suggesting a predominant acidity among XIP. Subcellular localization predictions revealed that 15 XIP genes were situated in the cell wall, 33 in extracellular, and 30 in vacuole, respectively. XIP-4A1 and XIP-4D1 localized in the Cell wall/Extracellular, XIP-3A3, XIP-3B3 and XIP-3D3 localized in the chloroplast/nucleus (Table S2). Signal peptide prediction indicated that 13 XIP lacked a signal peptide structure, while the signal peptides of the remaining XIP varied in length, ranging from 21-42aa.

**Fig. 1:**
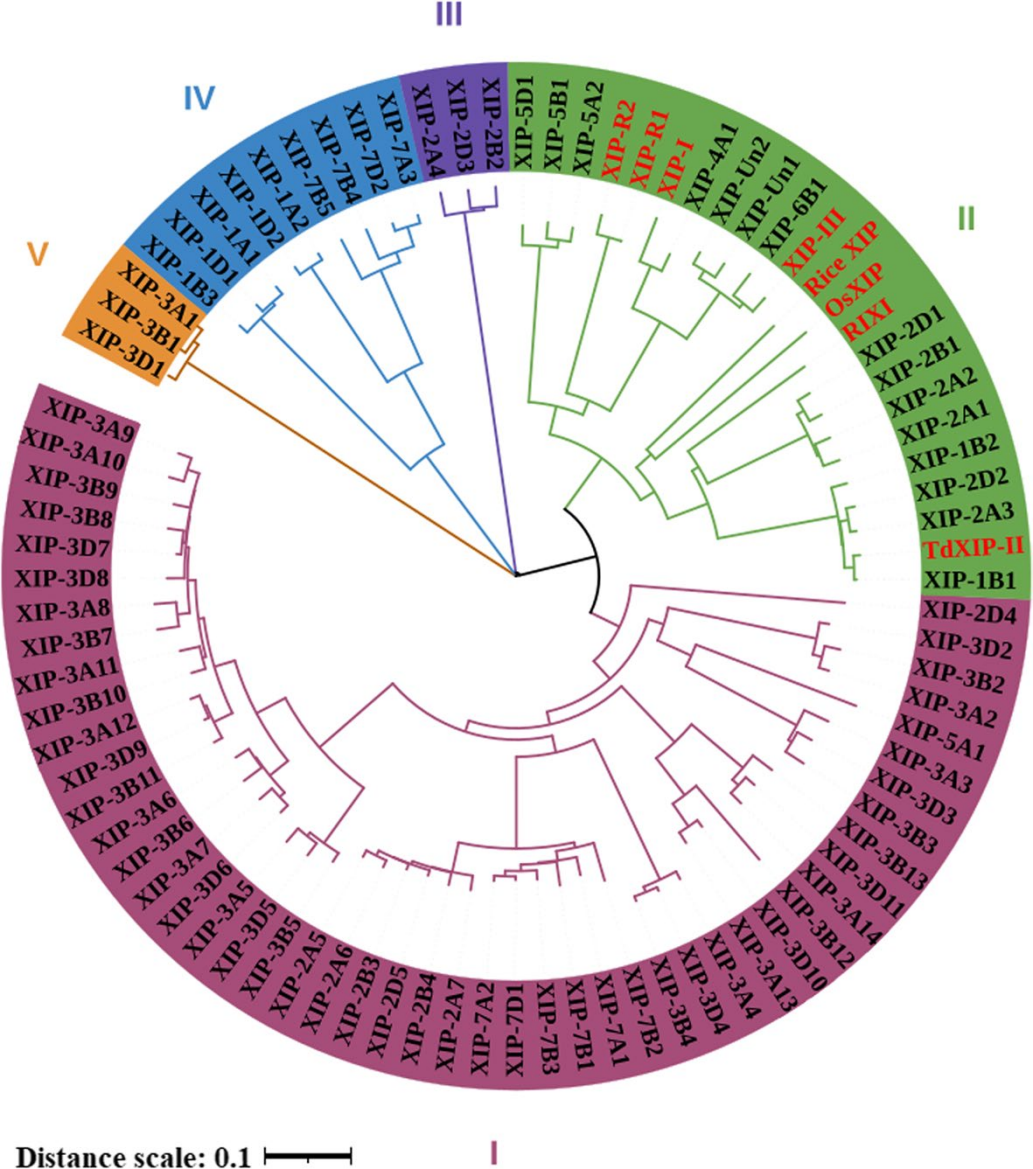
Phylogenetic tree of XIP genes. The tree includes 87 XIP genes comprised of 83 XIP family members from wheat, which include the previously reported wheat XIPs (XIP-I, XIP-III, XIP-R1 and XIP-R2), three rice XIPs (Rice XIP, OsXIP and RIXI), and one durum wheat XIP (TaXIP-II). Previously reported XIP genes are highlighted in red font, while the newly predicted wheat XIPs from this study are in black font. Different background colors categorize the XIP gene family into distinct groups based on their topological structure. ‘Un’ represents XIP genes not mapped to any chromosome

To understand the evolutionary relationships, we constructed a phylogenetic tree that encompassed 87 cereal crop XIPs, including 83 from wheat identified in this study, three from rice (Rice XIP, OsXIP and RIXI) and one from durum wheat (TaXIP-II). Five groups were resolved, with 49 XIPs belonging to group I, 23 to group II, 3 to group III, and 9 and 3 to groups IV and V respectively (Fig. 1). The XIP-type proteins from all examined cereal crops clustered within group II suggesting that genes within group II exhibit higher conservation across gramineous plants.

### Gene structure and protein conserved motifs analysis

Comparison with the NCBI conserved domain database revealed all XIPs to be part of the GH18 family. Notably, the GH18-related structural domains make up a large part of these XIP gene sequences (Fig. S1) implying that the characteristics that define the GH18 superfamily are a prominent feature in the structure and possibly the function of these XIP proteins.

**Fig. 2:**
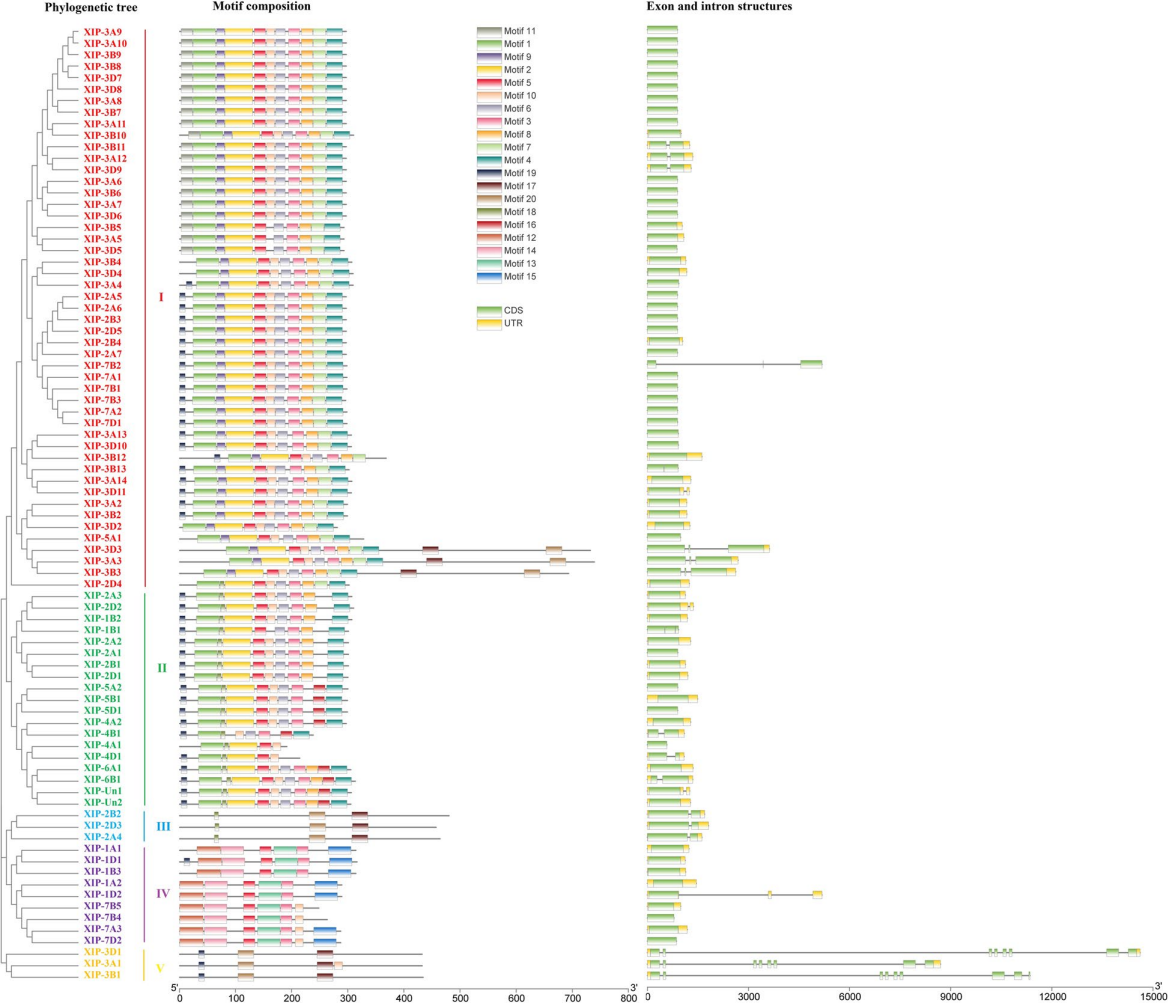
Comprehensive view of the wheat XIP gene family depicting conserved protein motifs and gene structure. Colored labels of the phylogenetic tree denote different XIP grouping. Adjacent to the tree is the motif composition of wheat XIP genes, with 20 distinct motifs represented by unique colors. Gene structure of the XIP family is featured with green boxes representing coding sequences, yellow boxes for untranslated regions, and black lines for introns. The protein and gene lengths are scaled at the bottom of the figure

In our analysis, we identified 20 distinct motifs across the 83 XIPs, designated as motif 1 to motif 20 (Fig. 2), with their sequence information detailed (Table S3). Certain motifs were consistently present across a majority of the XIP family members. For instance, most XIPs in group I and II exhibited motifs 1-6, 8, and 10. Each group displayed specific conserved motifs, suggesting a structured motif distribution. Group I XIPs uniquely possessed motif 7 and included motif 9 except for XIP-2D4. In contrast, group II XIPs exclusively featured motif 1. Group III XIPs possessed only motifs 17, 18 and 20, while all group IV XIPs contained motifs 3, 5, 12, 13 and 14. Group V XIPs predominantly showcased motifs 17, 19 and 20. This motif distribution hints at evolutionary divergence among XIP, which might influence the diversity of their functions.

In our gene structure analysis, we found that all XIP genes were structurally complete with a complete open reading frame, indicating they have the potential to encode fully functional proteins. However, 30 XIP genes in group I, five in group II, and two in group IV were observed to lack untranslated regions, as illustrated in Fig. 2. Notably, 65 out of 83, (78.31%) XIP genes comprised a single exon. The combined analysis of the gene structures and protein motifs highlights evolutionary relationships among these XIP genes, further corroborating their phylogenetic relationships.

### Collinearity analysis of XIP genes

**Fig. 3:**
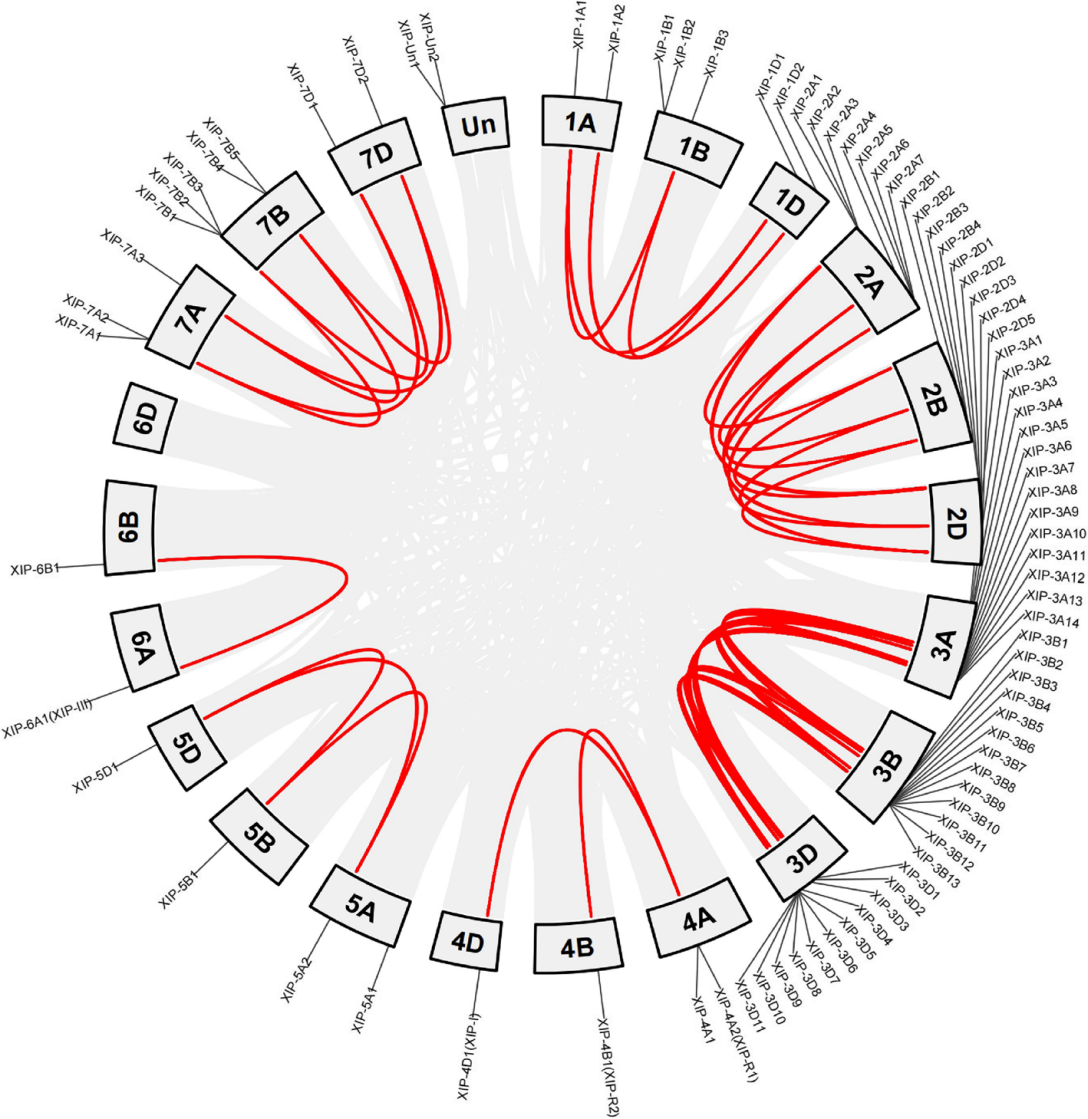
Segmental duplication of XIP gene pairs in the wheat genome. This figure displays collinear regions of the wheat genome connected by gray lines. The bold red line highlights the fragmentary duplicated pairs of XIP genes between homologous chromosomes, indicating their locations and relationships within the genome. Thickness of the red lines represents the relative frequency of collinear gene pairs between chromosomes

Exploration of the chromosomal distribution of XIP genes revealed interesting patterns across the wheat’s genome. Except for chromosome 6D, XIP genes were distributed on all chromosomes. Two XIP genes namely, *XIP-Un1* and *XIP-Un1* were not mapped to any chromosome. The gene count and their respective locations varied across chromosomes (Table S2 and Fig. 3). Chromosomes 4B, 4D, 5B, 5D, 6A, and 6B, each harbored only a single XIP gene, whereas chromosomes 3A, 3B, and 3D had highest gene counts with 14, 13, and 11, respectively.

Within the wheat genome, we identified 81,537 collinear regions, which include regions with genes of unknown (Un) chromosomal locations, encompassing a total of 76,005 genes. Of the XIP genes, 56 were situated within these collinear regions forming a total of 50 gene pairs (Fig. 3). Interestingly, the two XIP genes of ‘Un’ chromosomal locations, *XIP-Un1* and *XIP-Un2* did not pair with other XIP genes on other chromosomes. Conversely, chromosomes 4B, 4D, 6A, and 6B each showcased a single collinear pair. This distribution suggests that the emergence of these 56 genes is likely due to a fragmentary duplication event. Specifically, homologous chromosome 3 harbors the highest number of collinear XIP gene pairs, suggesting a pronounced propensity for gene duplication events on this chromosome. Thus, gene duplication appears to have significantly influenced the evolutionary expansion of the XIP gene family in wheat.

### *Cis*-elemental prediction of XIP promoters

**Fig. 4:**
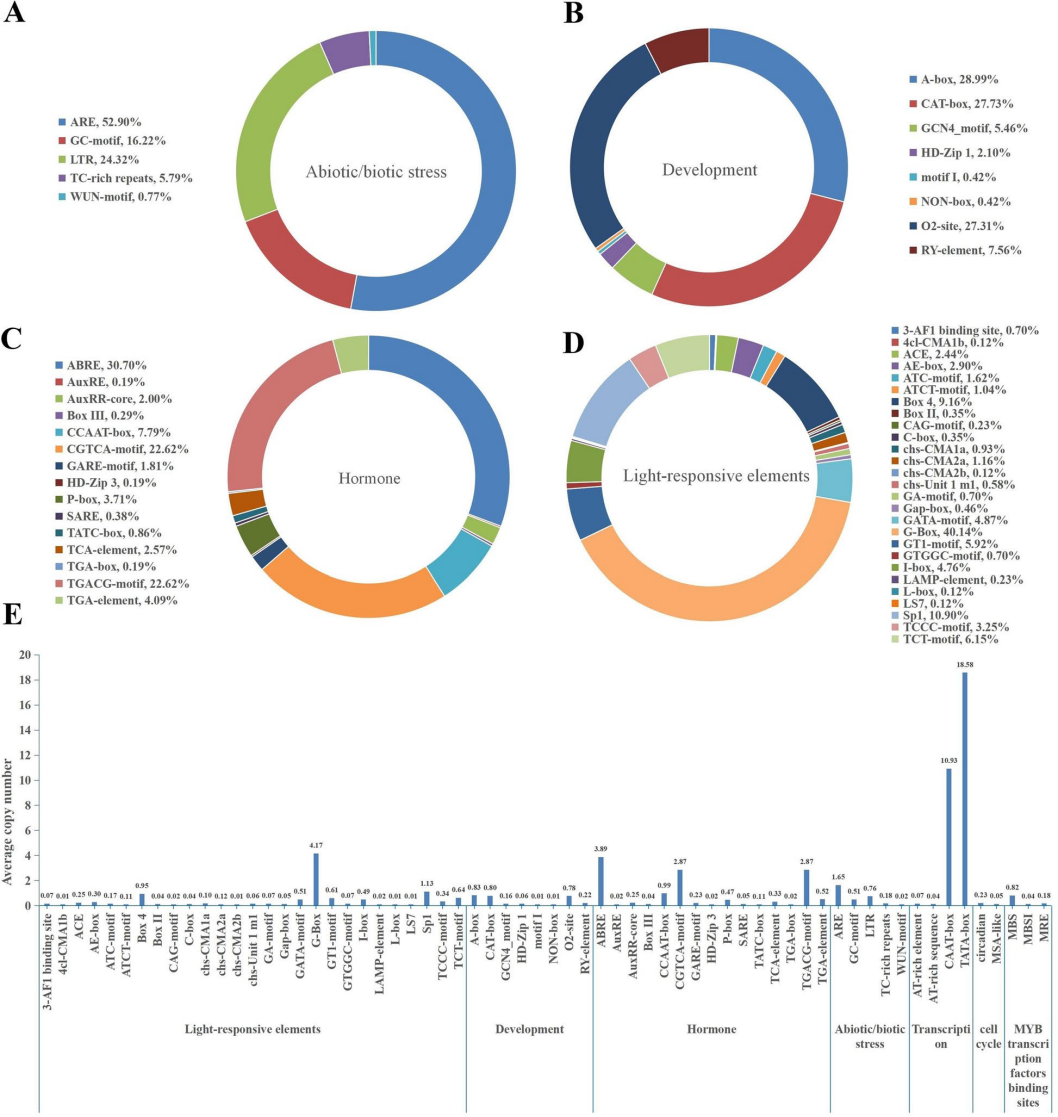
Comprehensive analysis of cis-acting elements in wheat XIP gene promoters. This figure illustrates the distribution and frequency of various cis-acting elements within the promoter regions of XIP genes. Panels (**A**) to (**D**) categorize these elements based on their functional roles: (**A**) abiotic and biotic stress response elements, (**B**) development-related elements, (**C**) hormone response elements, and (**D**) light-responsive elements. Each panel represents the proportion of specific cis-acting elements within its category. **E** displays the average occurrence of each cis-acting element across the XIP gene promoters

Scrutiny of the wheat XIP gene promoter regions revealed insights into understanding their role in development (Fig. 4B) and stress response (Fig. 4E). Within the promoter regions of XIP genes, all except for the *XIP-2B2* gene contained the fundamental cis-acting elements CAAT-box and TATA-box, with average occurrences of 10.93, and 18.58, respectively. Our comprehensive analysis revealed 64 distinct cis-acting elements that were associated with light response (Fig. 4D), hormone response (Fig. 4C) and both abiotic and biotic stress responses (Fig. 4A, Fig. S2 and Table S4).

The G-box, a light response element, was the most prevalent, appearing an average of 4.17 times across the 83 XIP genes, accounting for 40.14% of the light response elements. Hormonal response elements were also prominent. Most XIP genes contained ABA-responsive elements, ABRE (found in 81 genes) involved in ABA response with an average copy number of 3.89 and accounted for 30.7%. Similarly, CGTCA-motif and TGACG-motif elements (each found in 79 genes) associated with JA response were also predominantly found accounting for 22.62% with average copy numbers of 2.87 and 2.87, respectively. This suggests a potential role for most XIP genes in ABA and JA signaling pathways.

The anaerobic inducible response element, ARE (found in 73 genes), averaged 1.65 occurrences. Element related to tissue-specific expression, such as O2-site (found in 48 genes), A-box (found in 45 genes), and CAT-box (found in 44 genes), were observed with average occurrences of 0.78, 0.83, and 0.80, respectively. The presence of these cis-elements suggests that XIP genes might play roles in specific signaling pathways, modulating hormonal signaling and defense mechanisms against diverse stresses.

### Expression pattern of wheat XIP genes under stress of FHB infection

**Fig. 5:**
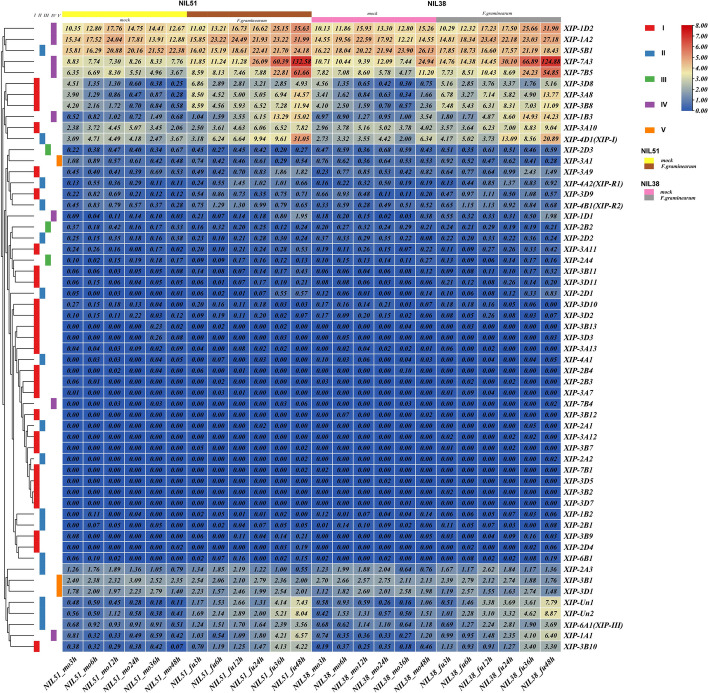
Differential expression of wheat XIP genes in response to FHB stress in Near Isogenic Lines NIL51 and NIL38. This heatmap illustrates the expression levels of XIP genes, measured in Transcripts Per Million (TPM) following FHB infection by *F. graminearum*, with data sourced from the WheatExp database. The expression values are presented on a log_2_-transformed TPM scale for enhanced clarity. The color bar reflects the abundance of transcription products, with different colors indicating varying levels of expression. The XIP genes are divided into five groups (I, II, III, IV, and V), each marked by distinct background colors. 'fu' indicates samples infected with *F. graminearum*, while 'mock' (mo) refers to control samples without FHB infection

To understand the XIP gene expression during FHB stress, we sourced expression data in Transcripts Per Million (TPM) value for wheat post-FHB infection from the WheatExp database [36, 37] (Table S5). This data was visualized using TBtools, employing a log_2_-transformation of the TPM value. The two lines, Near Isogenic Line 51 (NIL51) and NIL38 study were selected based on their publicly available detailed and accurate RNA-seq data pertaining to FHB. NIL38 has resistant alleles at both Fhb1 and Qhfs.ifa-5A which are among the most frequently studied resistance FHB QTLs [38], while NIL51 has susceptible alleles at both these QTL loci [33]. The heatmap (Fig. 5) shows the XIP gene expression in NIL51 and NIL38 at intervals of 3 h, 6 h, 12 h, 24 h, 36 h and 48 h post-FHB infection [33]. Inoculated plants were indicated as 'fu' FHB, while uninfected or control mock samples were labeled (mo). Notably, the expression patterns of XIP genes in both NIL51 and NIL38 under identical treatment conditions were strikingly similar. A handful of genes belonging to group I (*XIP-3D8*, *XIP-3A8*, *XIP*-*3B8*, *XIP-3A10*), group II (*XIP-5B1*, *XIP-4D1* (*XIP-I*)) and group IV (*XIP-1D2*, *XIP-1A2*, *XIP-7A3*, *XIP-7B5*, *XIP-1B3*, and displayed relatively higher expression at each staging post-FHB infection, generally surpassing mock (mo) levels in each timepoint. Both lines exhibited an increasing trend in the expression of these genes post-FHB infection, peaking at 48 h. Conversely, genes like *XIP-Un1*, *XIP-Un2*, *XIP-6A1* (*XIP-III*), *XIP-1A1* and *XIP-3B10* showed minimal expression across all stages, yet also demonstrated an increasing trend post-pathogen infection, reaching their zenith at 48 h.

### Candidate genes related to wheat FHB resistance

Using a screening criterion of TPM≥1 combined with either Fold Change (FC) ≥2 (up-regulated) or FC≤0.5 (down-regulated) [39], the gene expression was assessed across various timepoints studied after FHB infection. The fold change was calculated by comparing the expression in FHB-infected samples (fu) with mock-infected samples (mo) at corresponding time points. The comparison was made between mock and infected samples of a line at the same time point (e.g. NIL51_fu3h/NIL51_mo3h, NIL38_fu48h/NIL38_mo48h). A gene was considered a candidate if it met the screening criteria in any of these differential paired comparisons. The analysis identified twenty genes as potential candidates associated with FHB resistance in wheat, namely *XIP-1D2, XIP-1A2, XIP-7A3, XIP-7B5, XIP-3D8, XIP-3A8, XIP- 3B8, XIP-1B3, XIP-3A10, XIP-4D1 (XIP-I), XIP-3A9, XIP-4A2 (XIP-R1), XIP-3D9, XIP-4B1 (XIP-R2), XIP-1D1, XIP-Un1, XIP-Un2, XIP-6A1 (XIP-III), XIP-1A1, XIP-3B10*. Notably, all identified genes were up-regulated differentially, and no down-regulated genes were observed concerning FHB resistance. Detailed information on these genes is provided in Table 1.

**Table 1:** Candidate gene information table related to wheat FHB resistance

**Gene Name**	**Gene ID**	**Description**	**Amino Acid (AA)**	**Subcellular Localization**
*XIP-1A1*	*TraesCS1A02G141500.1*	Chitinase	314	Cell wall
*XIP-1A2*	*TraesCS1A02G284900.1*	Chitinase	289	Cell wall
*XIP-1B3*	*TraesCS1B02G158000.1*	Chitinase	314	Cell wall
*XIP-1D1*	*TraesCS1D02G140500.1*	Chitinase	316	Cell wall
*XIP-1D2*	*TraesCS1D02G283900.1*	Chitinase	289	Cell wall
*XIP-3A8*	*TraesCS3A02G373100.1*	Acidic endochitinase	297	Extracellular
*XIP-3A9*	*TraesCS3A02G373200.1*	Acidic endochitinase	297	Vacuole
*XIP-3A10*	*TraesCS3A02G373300.1*	Acidic endochitinase	297	Extracellular
*XIP-3B8*	*TraesCS3B02G405500.1*	Acidic endochitinase	297	Vacuole
*XIP-3B10*	*TraesCS3B02G405700.1*	Acidic endochitinase	310	Vacuole
*XIP-3D8*	*TraesCS3D02G366100.1*	Acidic endochitinase	297	Extracellular
*XIP-3D9*	*TraesCS3D02G366400.1*	Acidic endochitinase	297	Extracellular
*XIP-4A2 (XIP-R1)*	*TraesCS4A02G173800.1*	Dihydroxy-acid dehydratase	297	Extracellular
*XIP-4B1 (XIP-R2)*	*TraesCS4B02G143500.1*	Xylanase inhibitor protein 1	238	Extracellular
*XIP-4D1 (XIP-I)*	*TraesCS4D02G142000.1*	Xylanase inhibitor protein 1	214	Cell wall/ Extracellular
*XIP-6A1 (XIP-III)*	*TraesCS6A02G077000.1*	Xylanase inhibitor protein 1	305	Extracellular
*XIP-7A3*	*TraesCS7A02G371600.1*	Chitinase	287	Cell wall
*XIP-7B5*	*TraesCS7B02G256000.1*	Chitinase	248	Cell wall
*XIP-Un1*	*TraesCSU02G026200.1*	Xylanase inhibitor protein 1	306	Extracellular
*XIP-Un2*	*TraesCSU02G026500.1*	Xylanase inhibitor protein 1	305	Extracellular

Relative information such as description and amino acid (AA) can be found in the wheatomic platform (http://202.194.139.32). Subcellular localization was inferred using the Plant-mPLoc database on the Cell-PLoc 2.0 online platform (http://www.csbio.sjtu.edu.cn/bioinf/Cell-PLoc-2/)

### Promoter analysis and coding region homology analysis of candidate genes

**Fig. 6:**
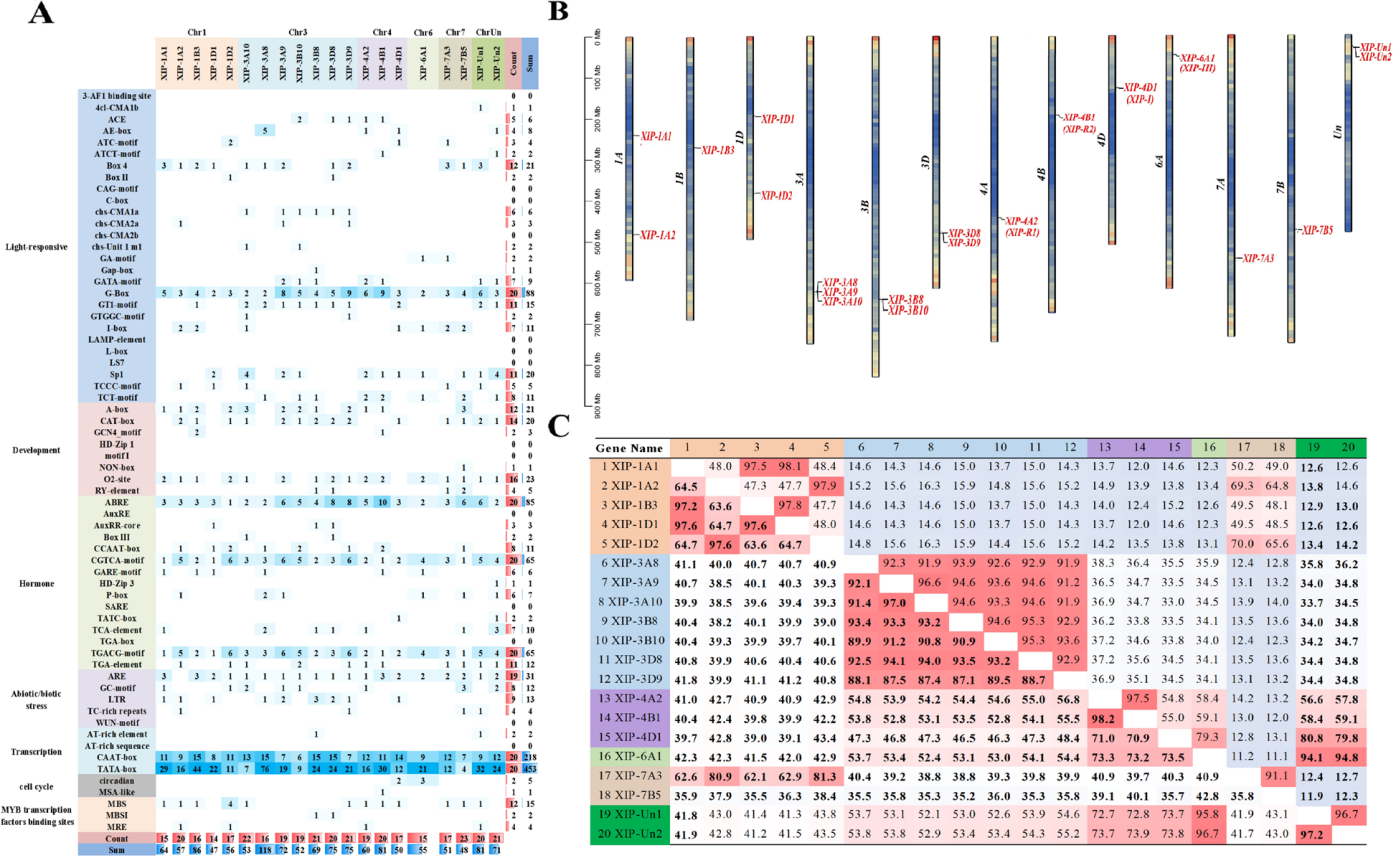
Analysis of 20 XIP candidate genes associated with wheat FHB resistance. **A** Distribution of various cis-acting elements in XIP genes. This chart displays the number of different cis-acting elements within the XIP genes, categorized into seven main types: Light-responsive, Development, Hormone, Abiotic/biotic stress, Transcription, Cell Cycle, and MYB Transcription Factors Binding Sites. A blank space indicates zero occurrences of an element. Different colors represent genes located on non-homologous chromosomes, while identical colors denote genes on homologous chromosomes. **B** Chromosomal localization of XIP genes. This part shows the position of XIP genes on wheat chromosomes, scaled in megabases (Mb). The labels 1A-7D represent the 21 chromosomes of wheat. **C** Sequence identity within XIP genes. The lower left section (in bold) shows the nucleotide sequence identity, and the upper right section displays the amino acid sequence identity. The identity percentages are calculated using the ClustalW method in MegAlign software

An examination of the cis-acting elements in the promoters of the 20 candidate genes revealed that all genes contained elements associated with ABA (ABRE) and JA (CGTCA-motif and TGACG-motif) responses (Fig. 6A). This suggests a potential role for these genes in disease resistance, possibly mediated through ABA and JA signaling pathways.

The identified candidate genes were found across various chromosomes: 1 (1A, 1B, 1D), 3 (3A, 3B, 3D), 4 (4A, 4B, 4D), 6A, 7 (7A, 7B). Some genes were also found in regions yet to be assigned to specific chromosomes, previously referred to as 'chromosome Un' (Fig. 6B). Notably, chromosomes 1B, 4A, 4B, 4D, 6A, 7A and 7B each harbored a single candidate gene. Chromosome 3 had the highest number of candidate genes with seven, followed by chromosome 1 with five.

A high degree of sequence homology was observed among the 20 candidate genes (Fig. S3). The results show that (Fig. 6C), genes on homologous chromosomes exhibited the most significant sequence similarity at both nucleotide (nt) and amino acid (aa) levels. For instance, genes on chromosome 1 showed 63.6-97.6% nt and 47.3-98.1% aa identity. Genes on chromosome 3 displayed 87.1-97% nt and 91.2-96.6% aa identity. And those on chromosome 4 had 70.9-98.2% nt and 54.8-97.5% aa identity. Interestingly, *XIP-Un1* and *XIP-Un2*, located on Un chromosomes, closely matched *XIP-6A1* in sequence, suggesting they might belong to homologous chromosome 6. A unique observation was the high amino acid identity (91.1%) between XIP-7A3 and XIP-7B5, despite a lower nucleotide identity (35.8%). Generally, genes on non-homologous chromosomes had higher nucleotide than amino acid sequence consistency. Overall, genes on homologous chromosome 3 exhibited the highest homology, followed by those on chromosomes 1 and 4.

### Functional annotation, pathway analysis, phylogenetic study and motif analysis of candidate genes

**Fig. 7:**
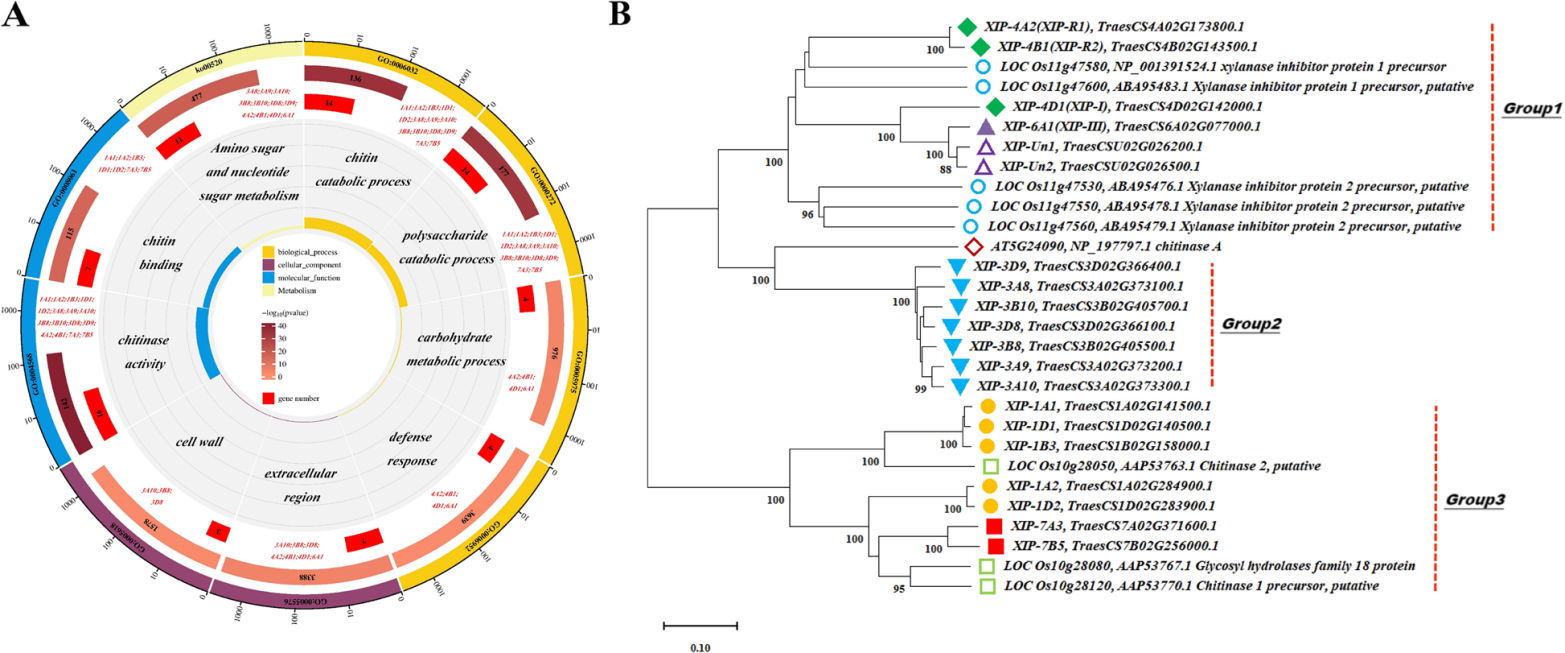
Comprehensive visualization of gene ontology and pathway enrichment. **A** Circle diagram of GO and KEGG pathway enrichment results. This circular diagram represents the outcome of the enrichment analysis. From the outer to inner layers, the diagram shows: Classification (with the same color indicating the same category, divided into biological process, cellular component, molecular function, and metabolism); the total number of genes enriched in each term across the genome (color variations represent different p-values of enrichment); the number of candidate genes enriched in each term; specific terms; and the rich factor (calculated as the number of candidate genes in the term divided by the total number of genes in the term). Enriched gene names are highlighted in red font. **B** Phylogenetic tree of homologous genes in wheat, rice, and Arabidopsis. In the phylogenetic tree, hollow blue circles and green squares represent rice genes from different chromosomes, and hollow red diamonds indicate genes from Arabidopsis. All other symbols, varying in color and shape, denote wheat genes, with each unique combination corresponding to genes from specific homologous chromosome groups in wheat

The shortlisted 20 XIP candidate genes underwent GO annotation revealing their involvement in various biological functions (Fig. 7A). Predominantly, these genes are associated with biological processes like chitin catabolic process (GO:0006032), polysaccharide catabolic process (GO:0000272), carbohydrate metabolic process (GO:0005975) and defense response (GO:0006952). They also exhibited molecular functions such as chitin binding (GO:0008061) and chitinase activity (GO:0004568). In terms of cellular components, they are primarily located in the extracellular region (GO:0005576) and cell wall (GO:0005618). The KEGG pathway enrichment analysis indicated that 11 genes were involved in the amino sugar and nucleotide sugar metabolism pathway (ko00520), which falls under the broader category of Carbohydrate metabolism (Fig. 7A).

To compare the evolutionary relationship of the 20 wheat XIPs with cereal crop XIPs, we used the wheatomic website to identify homologous genes in rice and Arabidopsis (Table S6). Most of these genes closely resemble XIPs and chitinase. Nine sequences, including one from Arabidopsis (AT5G24090) and eight from rice (LOC_Os10g28050, LOC_Os10g28080, LOC_Os10g28120, LOC_Os11g47530, LOC_Os11g47550, LOC_Os11g47560, LOC_Os11g47580 and LOC_Os11g47600), were retrieved from NCBI. Phylogenetic tree construction using NJ method (Fig. 7B) grouped these proteins into three distinct clusters: Group1, Group2, and Group3 (Fig. 7B). Group1 and Group2 XIP genes share homology with AT5G24090, while Group3 genes are homologous to specific rice genes like LOC_Os10g28050, LOC_Os10g28080, and LOC_Os10g28120. Notably, all wheat and rice proteins labeled as XIPs fall into Group 1, suggesting their close evolutionary relationship and potentially similar functions. Our analysis has identified several highly conserved motifs (motif 1 to motif 5) that are characteristic of the Glycoside hydrolase family 18 (Table S3 and Fig. S4).

### qRT-PCR analysis

**Fig. 8:**
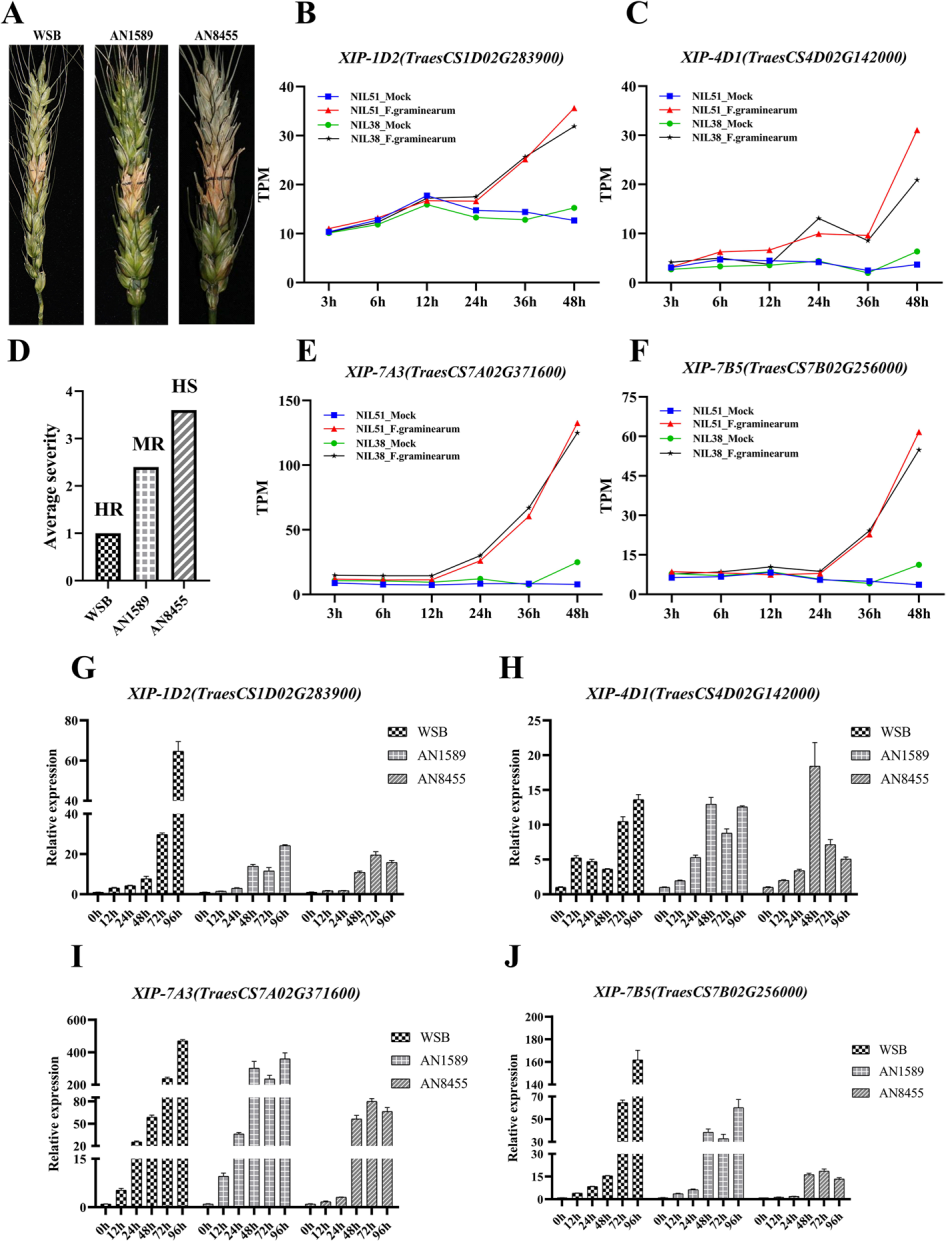
Differential expression of wheat XIP genes associated with FHB resistance. **A** Photographs of diseased wheat spikes taken 21 days after inoculation with *F. graminearum*. **D** Evaluation of disease severity. The average severity was calculated at 21 days after inoculation with *F. graminearum*. 'HR' denotes highly resistant cultivar, 'MR' for moderately resistant cultivar and 'HS' for highly susceptible cultivar. **B**, **C**, **E** and **F** Differential expression of wheat XIP genes in NIL51 and NIL38 in response to FHB stress. The expression levels of XIP genes were measured in Transcripts Per Million (TPM) following FHB infection, using data from the WheatExp database. Samples labeled as 'mock' refers to control samples without FHB infection. The figure displays the differential gene expression at various stages post-infection. **G**-**J** qRT-PCR analysis of XIP gene expression in response to FHB in three wheat cultivars. Differential expression of wheat XIP genes in response to FHB infection in Wangshuibai (WSB), Annong 1589 (AN1589) and Annong 8455 (AN8455), as measured by qRT-PCR

To delve deeper into the expression of XIP gene post-FHB infection and further validate the RNA-seq data, we carried out fluorescence quantitative experiments using artificial inoculation of three cultivars with contrasting levels of resistance to *F. graminearum*. Similar to the approach used in section 3.5, where resistant and susceptible NILs were analyzed using wheatomic data, we selected three wheat cultivars with varying resistance levels to FHB for our qRT-PCR experiments. The methodology, inoculation timing, and experimental conditions were meticulously aligned with those previously described to guarantee consistency across our experiments. Following the criteria established by other researchers, such as |logFC|>1 and TPM>10 for identifying and verifying candidate genes via qRT-PCR experiments [40], we focused our qRT-PCR experiments on *XIP-1D2*, *XIP-4D1*, *XIP-7A3* and *XIP-7B5*, which exhibited TPM≥20 (higher expression level) and FC≥2 in both NILs, thus minimizing the influence of Fhb1 and Qhfs.ifa-5A. Concurrently, we assessed the disease index (Table S7). The results revealed that WSB as a highly FHB-resistant cultivar, AN1589 as a moderately resistant cultivar and AN8455 as a susceptible cultivar (Fig. 8A and D). The expression trend of qRT-PCR aligned with that of RNA-seq data (Fig. 8). Throughout FHB infection, the expression of specific XIP genes varied based on wheat cultivars and infection durations. Analysis of the relative expression of gene *XIP-7A3* (*TraesCS7A02G371600*) and *XIP-7B5* (*TraesCS7B02G256000*) indicated an upward trend in expression with FHB infection, with resistant cultivars (WSB and AN1589) showing higher trends than the susceptible (AN8455). At 96 h after FHB infection, the expression trend of *XIP-1D2* (*TraesCS1D02G283900*) and *XIP-4D1* (*TraesCS4D02G142000)* was higher in resistant cultivars generally showing an upward trend in the process of FHB infection. In particular, the expression level of gene *XIP-4D1* in AN8455 gradually decreased after 48 hours of FHB infection. These findings suggest that XIP genes might be inducible by *F. graminearum*, corroborating the accuracy of RNA-seq data. Particularly, the expression of *XIP-7A3* (*TraesCS7A02G371600*) showed the most rapid increase among the studied genes, with expression levels in the WSB and AN1589 cultivars with approximately 470 and 360-fold changes, respectively, from 0 to 96 hours, post-infection (Fig. 8G-J). This significant upsurge in expression, far surpasses changes observed in other genes, suggesting *XIP-7A3* as a prime candidate gene associated with FHB resistance.

## Discussion

With the accumulating wheat genome-wide information, advancement and deployment of wheat genomic resources, and the development of bioinformatics software tools, comprehensive identification and analysis of the wheat XIP gene family is now possible. The use of comparative genomics approaches has been instrumental in discovering novel genes in cereals [41, 42], and the well-characterized wheat XIP-I serves as a valuable reference for identifying homologs in other cereal species. While extensive analysis of XIP-type genes in rice, maize, sorghum and short-stalked ferns have been conducted to understand their distribution and organization [43], a comprehensive identification of XIP genes in wheat has not yet been reported. In this study, we identified 83 XIP family genes, divided into five distinct groups. These genes are distributed across all wheat chromosomes except chromosome 6D (Fig. 1 and Table S2).

Our bioinformatic analysis, based on the amino acid composition, indicates distinct subcellular localizations for the XIP gene groups in wheat. Specifically, most XIP genes belonging to group I were predicted to localize in the vacuole or extracellular space, group II XIPs in the extracellular space, and all group III, IV, and V XIPs in the cell wall (Table S2). The cell wall serves as the primary defense barrier for plant cells against foreign pathogens, such as bacteria and fungi [44] potentially preventing their entry and subsequent damage to the cell. Additionally, the extracellular space, rich in molecules for intercellular communication and environmental sensing, [45] may facilitate XIPs in detecting pathogen invasion and triggering defense responses. The presence of XIPs in vesicles, which are crucial for intracellular substance transport and secretory reactions [46] suggests their potential accumulation and/or transport to the cell wall or extracellular space through secretory mechanisms, playing a defensive role against pathogens. As xylanase inhibitors, XIPs are known to bind and inhibit xylanases from pathogens making their subcellular localizations and secretion signals vital for their biological function [13].

Interestingly, 13 XIPs lack signal peptide structures, with 10 of these predicted to act on the cell wall or extracellular space (e.g., one XIP gene of group I in the extracellular space, six of group IV and three of group V in the cell wall) (Table S2). Signal peptides typically guide proteins to subcellular organelles or direct them into the endoplasmic reticulum for processing and eventual secretion [47, 48]. The absence of signal peptide structures in these XIPs suggests an alternative secretion pathway, possibly bypassing the conventional endoplasmic reticulum-Golgi apparatus route [49, 50]. This could result in faster secretion, either by direct translocation across the cytoplasmic membrane or by direct transport from the endoplasmic reticulum to the cytoplasmic membrane, leading to rapid accumulation in the cell wall or extracellular space [51]. Such mechanism could be critical for their role in combating disease. Notably, candidate genes *XIP-1D2*, *XIP-7A3* and *XIP-7B5*, all lacking signaling peptides, may exhibit a rapid response to FHB.

Intriguingly, most XIP genes are devoid of introns (Fig. 2), a characteristic consistent with previous findings in other species [43]. Studies have shown that genes with fewer introns can be rapidly induced by upstream signals [52], as the absence of introns allows for quicker transcription into mRNA by bypassing the RNA splicing step [53]. This feature may enable XIP genes to respond swiftly to environmental changes or signals, such as FHB infection, thereby, facilitating a rapid defense against pathogen attack.

Our study also reveals evidence of gene duplication in the wheat XIP gene (Fig. 3). Gene duplication, particularly in polyploid species, is known to play a crucial role in the evolution of resistance or tolerance mechanisms [54]. Given the increasing frequency and recurrence of wheat FHB, partly due to changes in farming systems and global climate [55], such duplication events can lead to an abundance of duplicated genes in plant genomes, promoting functional divergence or the acquisition of new functions in response to environments challenges [54]. The abundance of duplicated wheat XIP genes can be a reservoir for sub- and/or neo-functionalization which may have contributed to the acquisition of resistance mechanisms against FHB infection.

Furthermore, we observed a tendency for XIP genes to cluster on chromosomes (Fig. 3), aligning with findings in other species. Sonah et al. [43] suggested that gene clustering of XIP genes in Brachypodium, sorghum, maize and rice, might have resulted from horizontal transfer from prokaryotes. Horizontal gene transfer, occurring during long-term interactions between plants and their pathogens (e. g., bacteria or fungi), could lead to the acquisition of new traits to counteract the pathogens [56]. The clustering of genes on chromosomes may represent an adaptive trait, allowing for synergistic regulation in response to environmental changes [57]. Gene clusters are more common in fungal genomes compared to plants and higher animals [58], and previous studies have shown similarities between *XIP-I* like genes and fungal chitinase genes [43, 59, 60]. This raises the possibility that horizontal gene transfer from fungi might have contributed to the clustering of XIP genes in wheat. However, the specific origins and details of such transfer remain to be elucidated.

Cis-elements in the promoter region play a crucial role in regulating gene expression, and the types of cis-elements present can reflect the gene’s function [61]. The abundance of light-responsive cis-elements in XIPs (Fig. 4 and Table S4) suggests a potential involvement of these genes in photosynthesis. These elements could interact with transcription factors under varying light conditions, including changes in light intensity, quality and photoperiod [62], leading to either activation or repression of XIP gene expression. For instance, Li et al. [63] demonstrated in *Arabidopsis thaliana* that the photomorphogenesis transcription factor HY5 directly binds to the G/C hybrid element on the microRNA163 promoter. This binding triggers the light-responsive expression of microRNA163 inhibiting the accumulation of its target gene mRNA, and ultimately mediating taproot growth in response to light [63]. While this observation suggests that XIP genes might be regulated by light, further studies are warranted to investigate whether XIP genes are indeed light regulated and how light affects the function of these genes.

In addition to light-responsive elements, a significant proportion of XIP genes contain cis-elements associated with hormone responses (Fig. 4). Notably, out of 83 XIP genes, 81 (98%) possess ABRE cis-acting elements associated with the ABA response, and 79 XIP genes (95%) contain CGTCA-motif and TGACG-motif cis-acting elements associated with the JA response (Fig. 4 and Table S4). ABA, often referred to as the “stress hormone,” plays a vital role in plant adaption to abiotic stress through mechanisms such as stomatal closure, maintenance of osmotic balance, and regulation of the expression of stress-responsive genes [64–66]. Importantly, these functions are also extended to the context of biotic stress response. Stomatal closure can limit pathogen entry, maintenance of osmotic balance is vital for cellular integrity against pathogen-induced stress [67, 68], and the activation of stress-responsive genes can enhance plant defenses against various pathogens [65, 69]. Cheng et al. [70] demonstrated that exogenous application of ABA on tomato leaves significantly up-regulated the expression of disease resistance-related genes, including chitinase, as well as salicylic acid, ethylene, and JA signaling pathways [70]. Furthermore, studies have shown that in wheat spikes infected by *F. graminearum*, genes involved in JA biosynthesis and those regulated by JA signaling factors were up-regulated with a consequent increase in JA levels [71, 72].

Upon examining the distribution of XIP candidate genes in wheat, it becomes evident that these genes are predominantly situated at the terminal regions of chromosomes. This strategic positioning may facilitate the expansion of the wheat XIP gene family. Genes located at both ends of chromosomes are more prone to undergoing frequent recombination, which can be a significant source of genetic diversity [73]. This genomic feature is crucial for the expansion of gene families enabling plants to adapt more effectively to challenges, such as FHB infection [73]. The concentration of candidate genes for FHB resistance chromosomes 1, 3, 4, 6 and 7 (Fig. 6) in wheat, underscores the important role of polyploidization in gene family expansion [54, 74].

In this study, we observed that the genes XIP-7A3 and XIP-7B5 exhibit high amino acid homology but low nucleotide sequence homology (Fig. 6C). This phenomenon can be attributed to the degeneracy of the genetic code, which allows different nucleotide triplets or codons to encode the same amino acid [75]. This feature of genetic code enables significant evolutionary variations at the DNA level while conserving essential protein structure and function [76]. Such an evolutionary strategy ensures the critical functions of proteins are maintained, reflecting purifying selection pressures that preserve vital protein function against environmental stresses, including FHB infection.The Gene Ontology (GO) cellular component and KEGG pathway analysis align with our subcellular localization predictions for the candidate genes (Fig. 7A), indicating a strong similarity to chitinase (Fig. 7B). These genes predominantly exhibit molecular functions related to chitin binding and chitinase activity (Fig. 7A). Plant chitinases, classified as pathogenesis-related proteins [77], have been shown to enhance resistance to various pathogens, such as wheat powdery mildew and wheat FHB, particularly through the introduction of barley chitinase gene into wheat [77, 78]. During fungal invasion, maize chitinase hydrolyzes the chitin in fungal cell walls into chitin oligosaccharides [77, 79], which are then recognized by cell membrane receptors, triggering an immune response against the pathogen [77, 80].

Motifs analysis showed that 20 candidate genes have glycoside hydrolase family GH18-related conserved motifs (Table S3 and Fig. S4). GH18 is known for its catalytic activity in degrading chitin—a critical component of fungal cell walls [81, 82]. This suggests a significant role for these motifs in FHB resistance, as they are involved in the breakdown of pathogen structures and potentially inhibit xylanase, contributing to antifungal defense mechanisms.

Plant chitinases belong to glycoside hydrolase families GH18, GH19 and GH20 [77]. McLauchlan et al. [16] noted that structural similarities between XIP-I resembles GH18 chitinase, despite XIP-I lacking chitinase activity. Sonah et al. [43] hypothesized that XIPs might have evolved from chitinases, potentially acting not only against pathogen-secreted xylanase, but also directly on the pathogens themselves, given the rapid evolution and high sequence homology chitinases with XIPs. This hypothesis is supported by genome analyses showing high sequence similarity between XIPs and Chitinases [13], consistent with our findings. These analyses suggest that XIPs may have originated from functional diversification within the chitinase gene family [13]. Further experimental research is needed to ascertain whether XIP genes identified in this study possess chitinase activity and can inhibit the xylanase secreted by *F. graminearum*.

Our qRT-PCR experiments provide evidence that certain XIP genes are inducible in response to FHB infection in wheat. For this study, we selected Wangshuibai, a Chinese landrace known for its resistance to FHB [83] and two wheat cultivars developed by Anhui Agricultural University, Annong 1589 and Annong 8455. Annong1589, a recently developed cultivar, is notable for its high yield and moderate resistance to several diseases, such as Fusarium head blight (FHB), yellow rust, leaf rust, powdery mildew, and sharp eyespot. In contrast, Annong8455 is characterized by its high susceptibility to FHB. Our findings revealed that the expression *XIP-4D1(XIP-I)* is induced during FHB infection, suggesting its involvement in the wheat FHB resistance response. However, Igawa et al. [84] reported that the *XIP-I* in the wheat cultivar Norin 61 had no transcriptional activity under FHB infection, as determined using the Northern blotting method [84]. The observed discrepancies could stem from two primary factors. Firstly, it could be attributed to differences in wheat cultivars, as different cultivars may exhibit varying *XIP-I* expression patterns during FHB infection (Fig. 8) [26]. Genotypic differences could also play a role in observed contradictory results in which the cultivars used by Igawa et al. possessed functional polymorphisms in XIP-1 and/or other transcriptional factors rendering it inactive even upon FHB infection. Additionally, the sensitivity of the quantification methods used could play a role; Northern blotting may not be suitable for detecting low levels of gene expression, potentially leading to undetected signals in cases of low messenger RNA concentration [85, 86]. Our findings indicate that *XIP-I* expression levels were relatively low (Fig. 8), which might be below the detection threshold of Northern blotting. In contrast, qRT-PCR offers a more sensitive and broader quantitative range, making it more suitable for detecting subtle gene expression changes [85, 86].

We performed PCR amplification of the four candidate genes (*XIP-1D2*, *XIP-4D1*, *XIP-7A3*, and *XIP-7B5*) in the wheat cultivars WSB, AN1589, and AN8455. The PCR results showed no significant differences across these cultivars. However, to capture more detailed genetic variations that could affect gene expression, we employed advanced high-throughput sequencing techniques. This more sophisticated approach revealed potentially significant variations such as a single nucleotide polymorphism in the promoter of *XIP-4D1* and multiple variations in the promoter (Fig. S5) and a conservative mutation in the coding region of *XIP-7A3*. These findings suggest possible regulatory effects on gene expression, which could influence the plant's response to FHB infection.

Moreover, our combined analysis of RNA-seq and qRT-PCR data revealed that *XIP-7A3 (TraesCS7A02G371600)* exhibited the highest relative expression value and the fastest expression growth trend in the resistant cultivars (Wangshuibai and Annong 1589) (Fig. 8), potentially playing a positive regulatory role in the FHB response. The specific functions and molecular mechanisms of this gene in wheat resistance to FHB require further investigation.

## Conclusions

This study marks a significant advancement in wheat genomics by identifying and classifying 83 XIP genes, revealing their potential role in enhancing resistance against FHB. Our findings highlight the unique distribution and possible alternative secretion pathways of XIP genes, suggesting their importance in plant defense mechanisms. Despite limitations such as the need for direct experimental validation of light-regulation and functional diversification within the XIP gene family, this research provides a foundational understanding of XIP genes in wheat. Future studies should focus on experimental validation and exploration of these genes in other cereal crops, paving the way for developing wheat cultivars with improved resistance to FHB and other pathogens. This comprehensive analysis of the wheat XIP gene family offers valuable insights and resources for enhancing crop resilience against fungal diseases. Stacking of potentially beneficial XIP genes in breeding populations, specifically, *XIP-4D1(XIP-I)* and *XIP-7A3* based on our results may be a strategy to mitigate FHB outbreaks.

### Supplementary Information

Supplementary Material 1. 

Supplementary Material 2. 

Supplementary Material 3. 

Supplementary Material 4. 

Supplementary Material 5. 

Supplementary Material 6.

Supplementary Material 7. 

Supplementary Material 8. 

Supplementary Material 9.

Supplementary Material 10. 

Supplementary Material 11. 

Supplementary Material 12. 

## Data Availability

All the supporting data are included within the article and its additional files.

## Abbreviations

XIP: Xylanase inhibitor protein
FHB: Fusarium head blight
F. Graminearum: Fusarium graminearum
XIs: Xylanase inhibitors
TLXI: Thaumatin-like xylanase inhibitor
TAXI: Triticum aestivum xylanase inhibitor
GH18: Glycoside hydrolase family 18
JA: Jasmonic acid
ABA: Abscisic acid
HMM: Hidden Markov model
NCBI: National Center for Biotechnology Information
MEGA11: Molecular Evolutionary Genetics Analysis11
bp: Base pairs
GO: Gene Ontology
KEGG: Kyoto Encyclopedia of Genes and Genomes
WSB: Wangshuibai
AN1589: Annong1589
AN8455: Annong8455
Aa: Amino acids
Un: Unknown
TPM: Transcripts Per Million
NIL: Near Isogenic Line
FC: Fold Change
Nt: Nucleotide
