# Supplementary figures and images for: Comprehensive genome-wide analysis of wheat xylanase inhibitor protein (XIP) genes: unveiling their role in Fusarium head blight resistance and plant immune mechanisms
Source: BMC Plant Biol. 2024 May 27;24:462. doi: 10.1186/s12870-024-05176-4 (PMC11129392; doi:10.1186/s12870-024-05176-4)

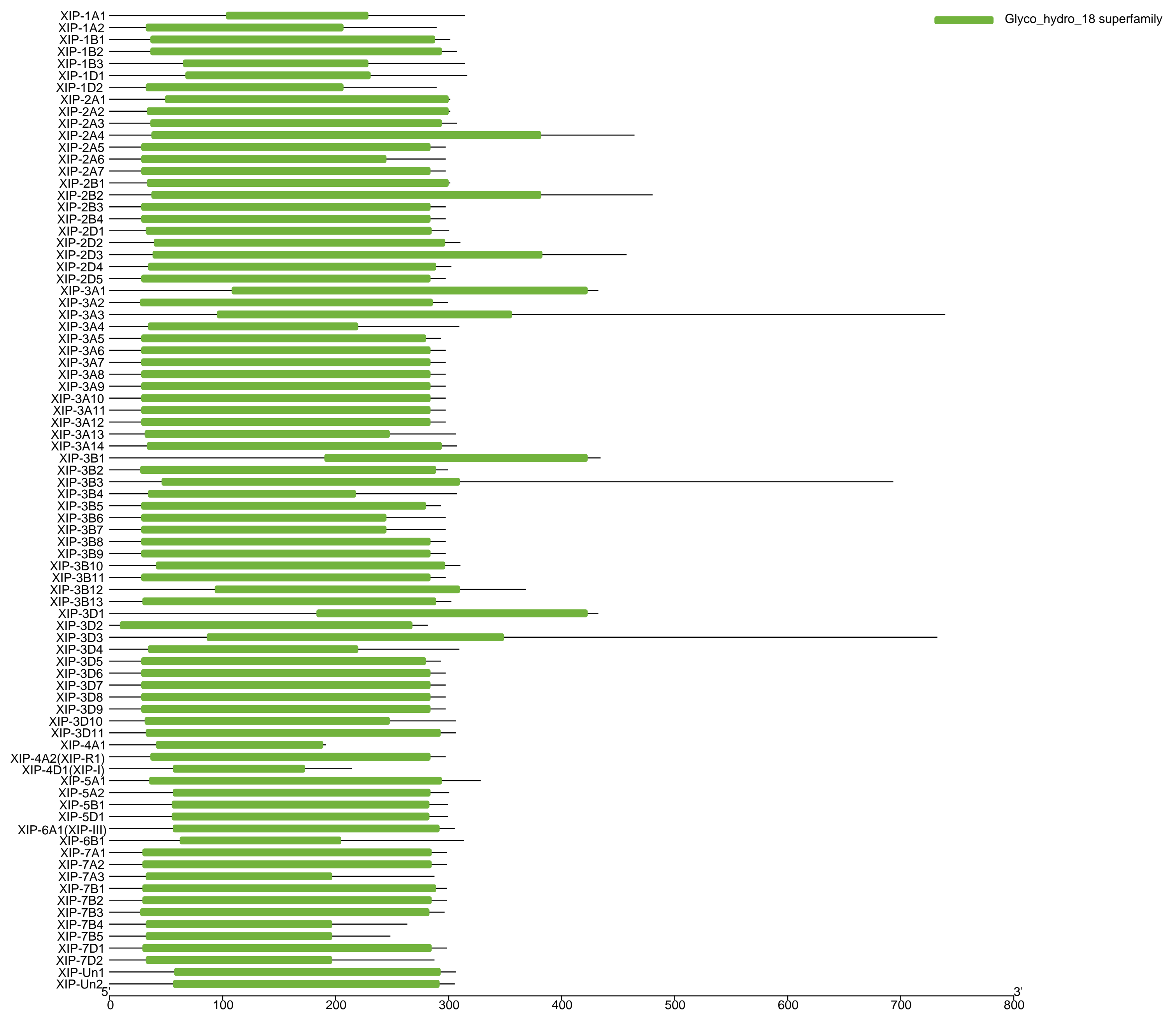

Supplement: Supplementary file 1 — Supplementary Material 1. [file 12870_2024_5176_MOESM1_ESM.pdf]

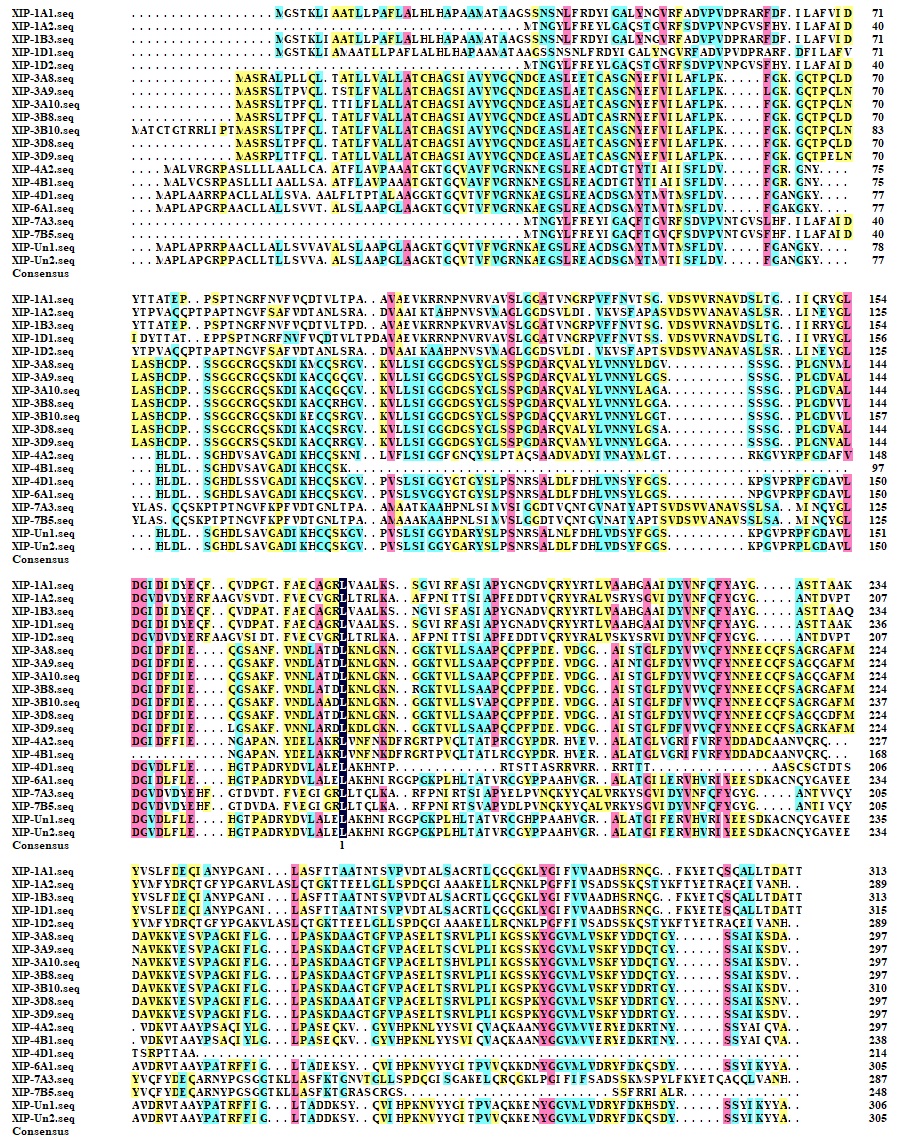

Supplement: Supplementary file 3 — Supplementary Material 3. [file 12870_2024_5176_MOESM3_ESM.jpg]

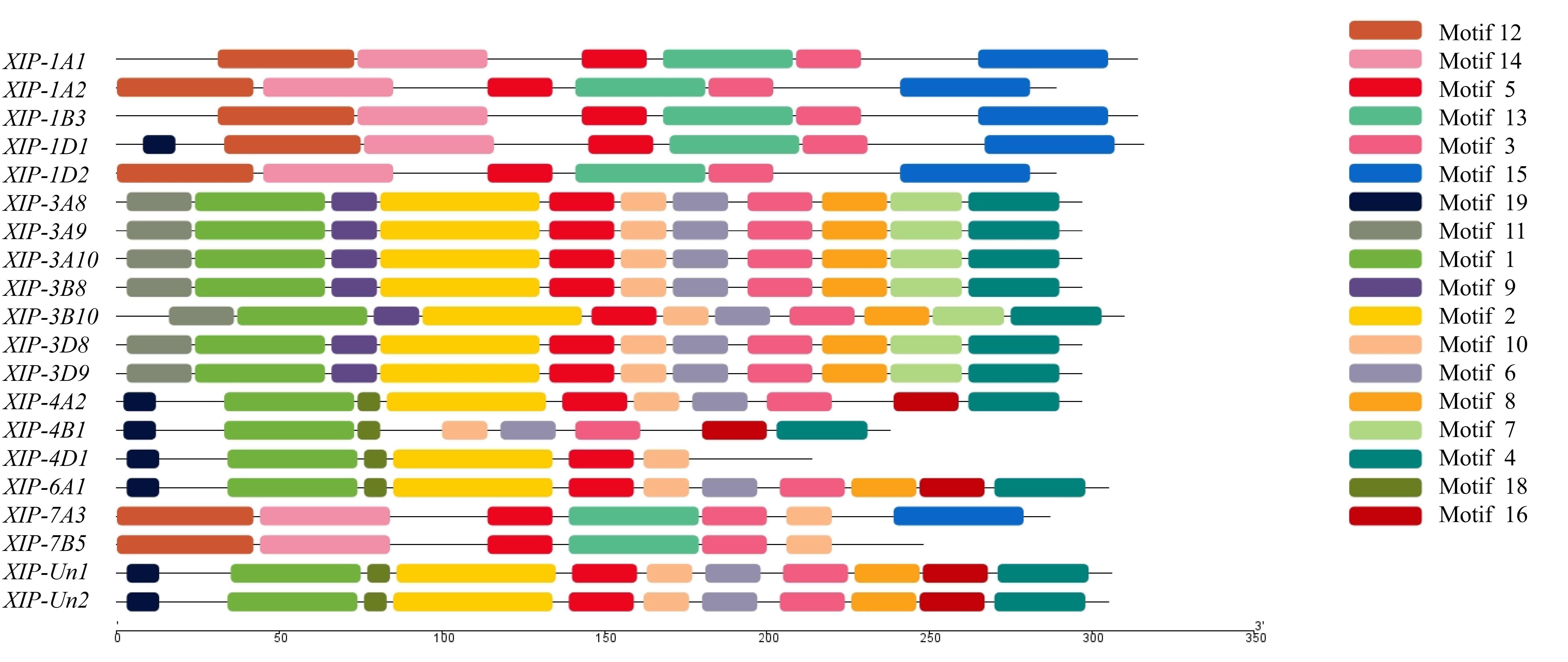

Supplement: Supplementary file 4 — Supplementary Material 4. [file 12870_2024_5176_MOESM4_ESM.jpg]

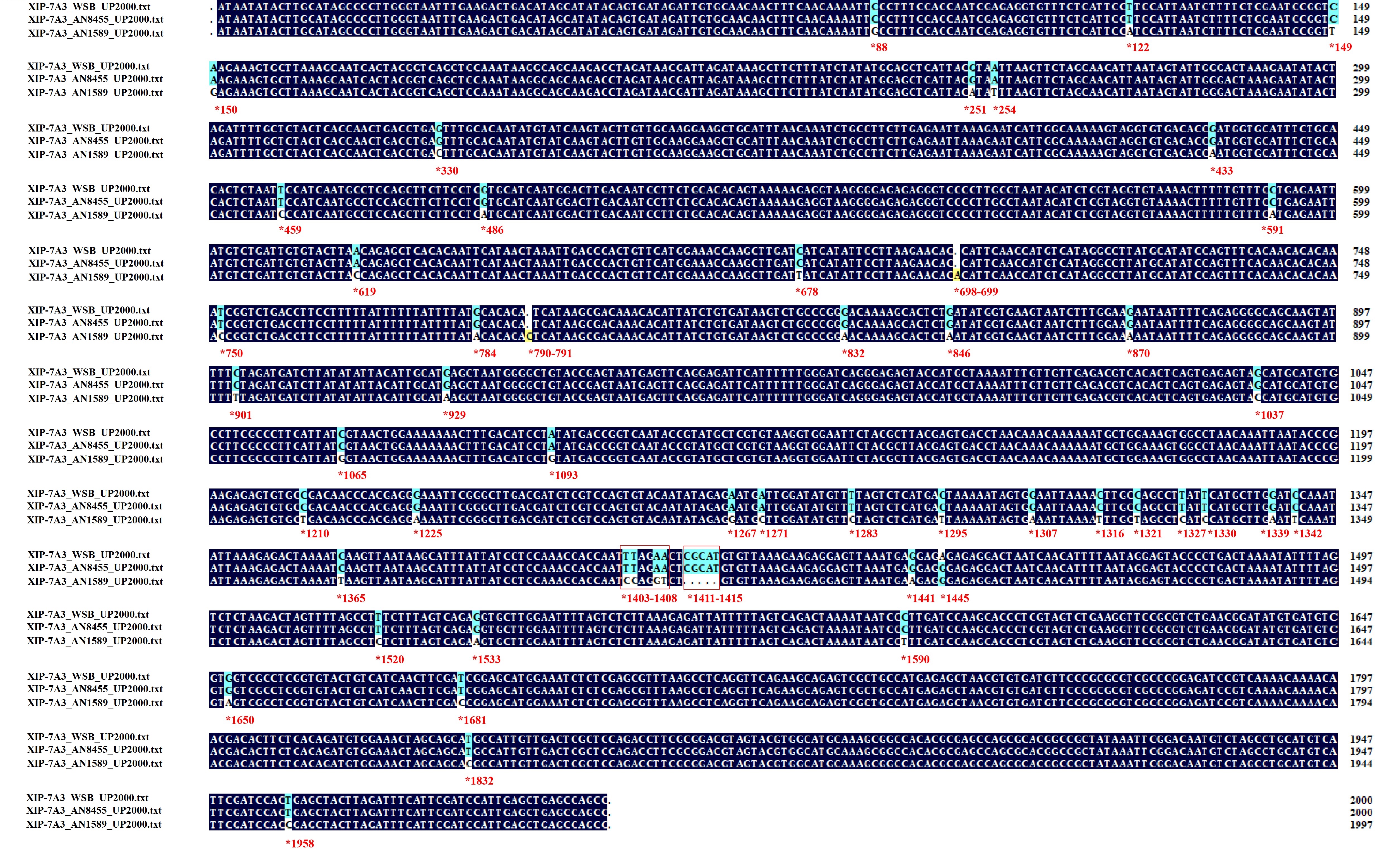

Supplement: Supplementary file 5 — Supplementary Material 5. [file 12870_2024_5176_MOESM5_ESM.jpg]
